# Polyacrylic Acid concentrations on biomimetic remineralization in artificial enamel lesions

**DOI:** 10.1186/s12903-026-07978-4

**Published:** 2026-02-28

**Authors:** Paul Vieweg, Maj Dieke, Marc Staiger, Sebastian Paris, Basel Kharbot

**Affiliations:** 1 Department of Operative, Preventive and Pediatric Dentistry, Charité – Universitätsmedizin, Aßmannshauser Str. 4-6, Berlin, 14197 Germany; 2 Physical Chemistry, Department of Chemistry, University of Konstanz, Constance, 78464 Germany

**Keywords:** Polyacrylic acid, Remineralization, Caries, Non-classical crystallization, Polymer-induced liquid precursor, Crystallization

## Abstract

**Background:**

The study aimed to evaluate the effect of Polyacrylic Acid (PAA) concentration on the remineralization of artificial enamel lesions in a pH cycling (pHc) model in vitro, as well as the effect of different durations to the pHc.

**Methods:**

Bovine enamel specimen were prepared and two artificial caries lesions were created with a demineralization solution (pH 4.95; 28 d) separated by varnish. Specimens were randomly assigned to three groups for different PAA concentrations (*n* = 40). One lesion in each specimen was etched (37% H_3_PO_4_, 5 s) and infiltrated with PAA (0.1, 1 or 10 mg/ml), the other lesion served as a non-treatment control (NTC). To obtain a baseline control (BL), specimens were cut perpendicularly to the lesions. The remaining halves (E) were exposed to pHc (21 h/d: pH = 7; 3 h/d: pH = 4.95) for either 28 d or 56 d (*n* = 20). The difference in integrated mineral loss between baseline and after pHc was analyzed using transverse microradiography (ΔΔZ = ΔZ_E—_ΔZ_BL_).

**Results:**

After 28 days, lesions treated with 0.1 mg/ml and 1 mg/ml PAA showed significantly greater mineral gain than NTC (*p* < 0.05, Wilcoxon), while 10 mg/ml PAA showed no significant effect (*p* > 0.05). Prolonging pHc to 56 days did not yield significant differences between groups (*p* > 0.05, Kruskal–Wallis).

**Conclusions:**

Lower concentrations of PAA seem to promote remineralization in the chosen pHc model.

## Background

Modern dentistry has shifted its focus to early intervention strategies for initial caries lesions, employing non-invasive and micro-invasive treatment measures [1–3]. Non-invasive interventions aim to prevent lesion formation or progression through dietary adjustments, biofilm control, and the topical application of fluorides. Although these interventions can be easily implemented in clinical practice with minimal risk, they often rely heavily on patient adherence, representing an inherent limitation. Furthermore, remineralization promoted by fluorides typically occurs primarily on the lesion surfaces, while the lesion body remains largely demineralized [4–7]. Various alternative approaches have been explored for the ‘biomimetic’ remineralization of early caries lesions, reaching also deeper lesion parts. Casein-phosphopeptide-amorphous calcium phosphate (CPP-ACP) for example aims to stabilize higher concentrations of Ca^2+^ and PO_4_^3−^ ions, while nano-hydroxyapatite serves as a synthetic substitute for dissolved minerals [8–10]. The self-assembling peptide P11-4 is an oligomeric β-sheet-forming peptide that can self-assemble into three-dimensional scaffolds. Due to its affinity for Ca^2+−^ions, it can act as a nucleator for de novo hydroxyapatite formation in enamel caries lesions [11–13]. Many of these approaches seek to either replace fluoride or to enhance its efficacy. While the remineralization potential of these approaches appears promising, long-term clinical evidence is lacking, and their efficacy, particularly in comparison to the conventional application of fluoride, remains unproven [14, 15].

An alternative approach to arrest non-cavitated lesions is caries infiltration. This micro-invasive intervention aims to create a diffusion barrier for cariogenic acids through the infiltration of the lesion body with a light-curing resin. This approach involves the removal of the hypermineralized surface layer with an acid and subsequent infiltration of the porous lesion with a low-viscosity resin, facilitated by capillary forces. Although the efficacy of resin infiltration is well documented [16–18] this approach is not biomimetic as the lost hydroxyapatite is replaced by resin and not by mineral again.

We recently introduced an approach that aims to combine the concepts of non- and micro-invasive strategies to deliver remineralizing agents rapidly to the lesion bodies of artificially created enamel caries lesions [19]. This approach seeks to address common limitations of current remineralization agents by exploring non-classical crystallization (NCC) pathways. In contrast to the layer-by-layer growth observed in classical crystallization processes, NCC involves the formation of crystals through the use of larger building units, such as nanoparticles or liquid droplets. One potential pathway includes the transformation of liquid droplets when stabilized with polyelectrolytes, such as polyacrylic acid (PAA), which have been previously described as polymer-induced liquid precursors (PILP) phases [20–22]. In this instance, the process utilizes the affinity between the negatively charged carboxylate groups of PAA and the positively charged Ca^2+^ ions to stabilize premature crystal units. The potential of PAA as a structuring and stabilizing unit has been demonstrated for collagen-containing hard tissues such as dentin and bone as well as for enamel in a pH-cycling (pHc) model [19, 23–25]. In the present approach, the concept of caries infiltration was adapted to rapidly penetrate the lesion body with PAA, driven by capillary forces. Subsequently, PAA was supposed to promote remineralization through the NCC pathway in subsurface areas in a pHc-model.

Both the composition and concentration of acidic polypeptides have been found to significantly influence their capacity to form PILP phases [26–31]. Therefore, the objective of this study was twofold: firstly, to evaluate the effect of varying PAA concentrations on the integrated mineral loss of enamel caries lesions in a pHc model, as well as the effect of different exposure durations to the pHc; secondly, to confirm the presence of PAA in the lesion bodies of treated lesions. The null hypothesis posited that, first, the concentration of PAA and, second, the duration of exposure to a pHc would not have a significant effect on the integrated mineral loss of artificial enamel lesions in vitro.

## Methods

### Study design

**Fig. 1:**
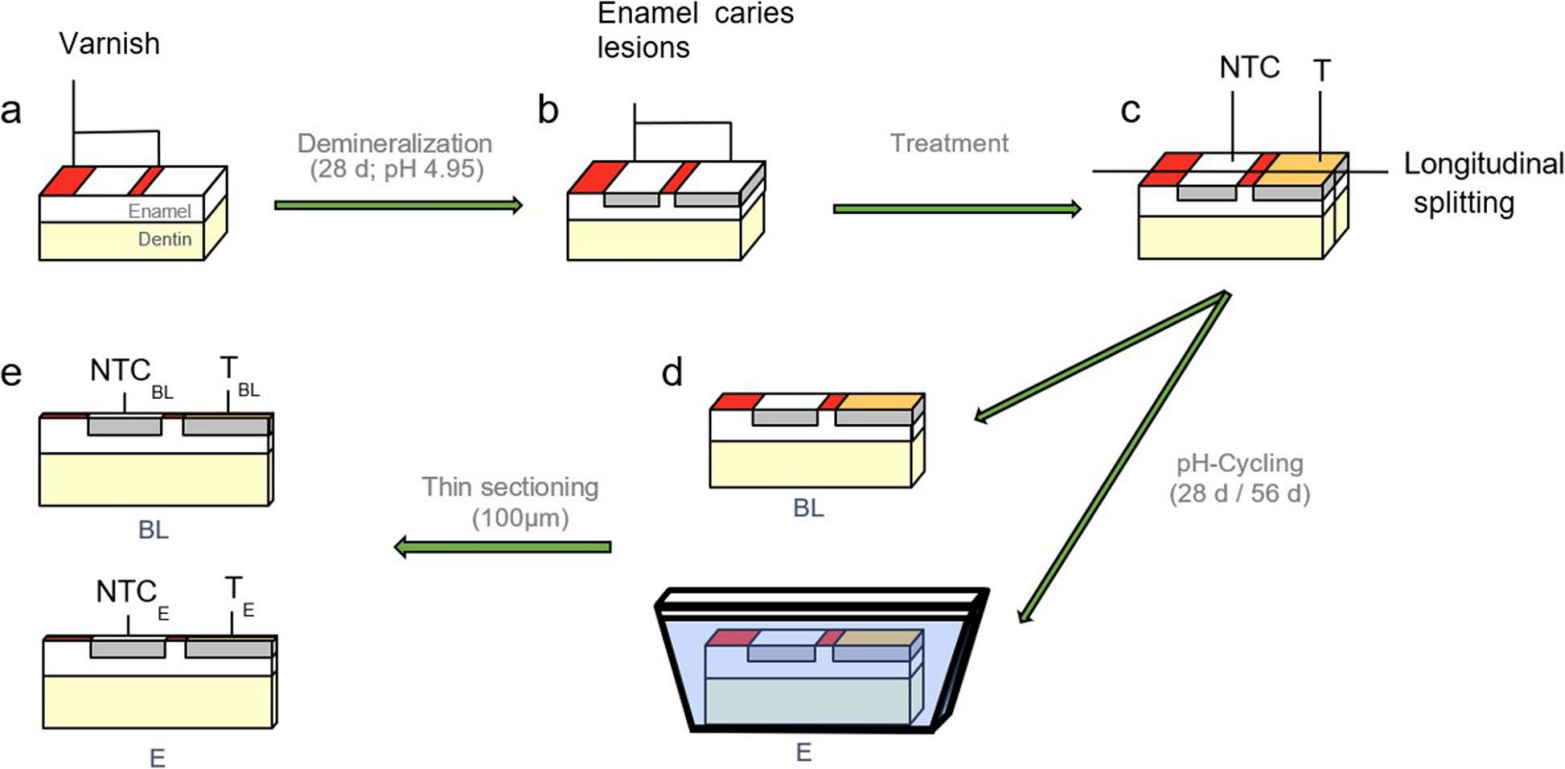
Schematic illustration of the sample preparation process. The bovine enamel specimens were covered with nail varnish to maintain a sound enamel area and to separate two enamel areas, non-treated controls (NTC) and treated (T) (**a**), in which caries lesions were induced (**b**). After the surface areas T were etched and infiltrated with the respective PAA solutions (**c**), each specimen was cut longitudinally into a Baseline (BL) and an Effect half (E) (**d**). The E halves were subjected to a pHc for 28 d or 56 d, respectively, prior to thin sectioning and TMR analysis (**e**)

A total of 120 bovine enamel samples were prepared with two artificially created carious lesions in each specimen (Fig. 1). The specimens were randomly allocated to 3 groups (*n* = 40). One lesion per specimen served as a no-treatment control (NTC), while the other lesion (T) was treated with PAA solutions with varying concentrations of 0.1 mg/ml, 1 mg/ml, and 10 mg/ml. After treatment, the specimens were cut perpendicularly to the lesions, thereby obtaining baseline control sections (BL) and effect sections (E). Final analyzes were performed with 17 specimens per group due to specimen loss during the sample preparation process. The BL sections were analyzed immediately, while the E sections of each concentration were exposed to a pH-cycling regimen for either 28 d or 56 d, respectively. Integrated mineral loss was analyzed using Transverse Microradiography (TMR) and changes in mineral loss (ΔΔZ) and lesion depth (ΔLD) between E and BL were calculated.

### Specimens preparation

Caries-free bovine incisors were extracted, cleaned and stored in chloramine-t solution (0.5%). The roots were separated and standardized specimens (5 × 9 x 3 mm^3^) were cut out of the labial aspect of the crown (EXAKT Trennschleifsystem 300 CL, Fa. EXAKT Vertriebs GmbH, Norderstedt, Deutschland) and ground flat (Labo Pol 25, Struers GmbH, Ballerup, Dänemark/Willich, Deutschland) under permanent water cooling. The enamel-dentin blocks were embedded in acrylic resin (Technovit 4071, Heraeus Kulzer, Werheim, Deutschland) and the enamel surfaces polished with grinding paper (Schleifpapier, SiC, grain size 1000–4000, Buehler GmbH, Düsseldorf, Deutschland) to create an even surface. Two areas of the samples then were covered with acid resistant nail polish (Maybelline Jade, Express Finish Nagellack, L'ORÉAL Deutschland GmbH, Düsseldorf, Deutschland) to obtain a sound enamel area (S) and two separate, uncovered enamel windows serving as NTC and T.

Artificial carious lesions were created by storing all specimens in a repository filled with 5 L of a demineralization solution containing 50 mM acetic acid, 3 mM CaCl_2_ ∙ 2H_2_O, 3 mM KH_2_PO_4_ and 6 µ Methylhydroxydis-phosphonate (pH 4.95; 37 °C) for 28 days [32]. The pH was monitored daily and adjusted with 10 M KOH or 1 M HCL, if necessary. All specimens were carefully rinsed with aqua dest. (Ampuwa® Spüllösung, Fresenius Kabi, Bad Homburg, Deutschland) and stored in humid chambers at 3 °C.

### Sample treatment

**Fig. 2:**
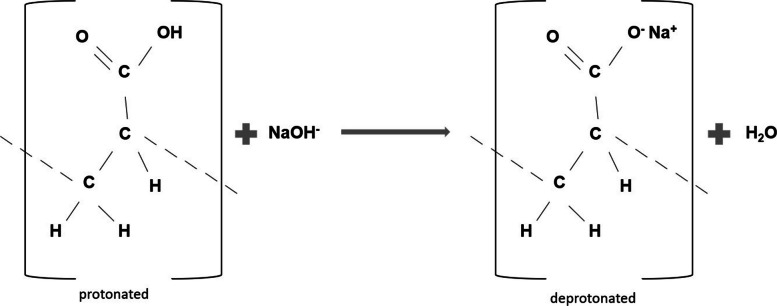
Chemical structure of the protonated Polyacrylic Acid and the deprotonated Polysodium Acrylate

PAA-solution (10 mg/ml) with a pKa of 4.5 was prepared by mixing 0.714 g of polyacrylic acid sodium salt (molar mass of 15000 g/mol; Sigma-Aldrich, St. Louis, MO, USA) with 17 ml deionized water (Fig. 2). The pH was adjusted to 9 using 0.1 M KOH. Parts of the solution were then diluted to concentrations of 1.0 mg/ml and 0.1 mg/ml with distilled water based on previous studies investigating mineralization potential in dentine and bone [29, 31].

One lesion of each specimen (T) was etched for 5 s with phosphoric acid (37%, ORBIS Dental Ätzgel) and rinsed off for 30 s to remove the pseudo-sound surface layer. After drying, the lesions were subsequently infiltrated by applying the respective PAA solution for 10 min. The specimens were cut (EXAKT Trennschleifsystem 300 CL, Fa. EXAKT Vertriebs GmbH, Norderstedt, Deutschland) to obtain two separate sections of each specimen (BL and E). The lesion surfaces of BL sections were coated with nail polish and stored in a humid chamber at 3 °C until TMR analysis. The E sections were coated on the cut surfaces to prevent penetration of fluids into the enamel before exposition to the pHc.

The pHc was conducted by storing all the E halves of each sample in a chamber containing 5 L each of the above described demineralization solution and a remineralizing solution containing 1.5 mM CaCl2·2H2O, 0.9 mM KH2PO4, 130 mM KCl, 20 mM hydroxyethylpiperazine-N’−2-ethanesulfonic acid (HEPES) buffer, 3 mM NaN3 and 5.26 mM F − (pH 7; 37 ◦C) [32] using an automatic, time-stamped pumping system (Peristaltic pumps P4, Seko, Rufina, Italy; EG-PM2 programmable power outlet strip, Energenie, Almere, The Netherlands) performing 3 h of demineralization periods and 21 h of remineralization periods per day. In order to avert inconsistent pH levels due to solution residues, short rinsing cycles with water were implemented between period changes.

### Transversal microradiography

Transversal Microradiography (TMR) was used for the quantitative analysis of integrated mineral loss (ΔZ) of the lesions. Specimens were attached (UHU dent; UHU GmbH, Bühl/Baden, Deutschland) on object holders (Plexiglas-Objektträger 25 × 75x2mm; Fa. Dia-plus, Oststeinbek, Deutschland) to prepare thin sections to a final thickness of 100 ± 10 µm with a grinding machine (EXAKT Mikroschleifsystem 400 CS, Fa.EXAKT Vertriebs GmbH, Norderstedt, Deutschland) and grinding paper (SiC, P 1000–4000, Buehler GmbH, Düsseldorf, Deutschland). The thin sections were mounted on custom made holders (Charité Facility Management GmbH, Mechanische Werkstätten, Campus Benjamin Franklin, Berlin, Deutschland) and x-rayed at 20kV and 10 mA with an exposure time of 10 s (PW 2213/20, Panalytical, Kassel, Deutschland). An aluminum stepwedge with 12 steps each with an increasing thickness of 25 µm was included for calibration. The x-ray films (Fine 71,337, FUJIFILM Corporation, Tokyo, Japan) were developed and ΔZ and LD were digitally analyzed using a microscope (Axioskop 2, Fa.Zeiss, Oberkochen, Deutschland) with a CCD-video camera module (XC 77 CE, Sony, Tokyo, Japan) and corresponding software (TMR for Windows, Version 2.0.27.2, Inspektor Research, Amsterdam, Niederlande) as described previously [33].

### Infrared microscopy (IRM)

To verify the infiltration of PAA into the lesion body, two-dimensional infrared microscopy (IRM) (Lumos FT-IR Microscope; Bruker Corporation, Billerica, MA, USA) was performed with three different specimens treated with 10 mg/ml PAA. As a reference, the PAA solution (10 mg/ml) was measured via IRM with a range of 4000 cm^−1^ – 600 cm^−1^ and a resolution of 4 cm^−1^ in reflection mode as well as attenuated total reflection infra-red (ATR-IR) spectroscopy (Cary 630 FTIR Spectrometer with ATR sampling module; Agilent Technologies Deutschland GmbH, Waldbronn, Germany) with a range of 4000 cm^−1^ – 600 cm^−1^ and a resolution of 2 cm^−1^ in transmission mode. The distribution of the intensity of a characteristic infrared (IR) active peak of PAA was compared between NTC and T lesions. A total of 21 points with an illumination area of 40 × 40 μm^2^ were measured throughout the lesion body (Fig. 8, indicated by green dots within red grids). The IR spectrum was obtained through the measurement of 256 scans, with a range of 4000 cm^−1^ – 600 cm^−1^, and a resolution of 4 cm^−1^ in reflection mode. The data were acquired and processed using the associated software (OPUS 7.5, Bruker). For each measurement point, an integral value from the corresponding IR spectrum was calculated over different wavenumber ranges. The integral was constructed using a baseline that was aligned with the spectrum itself at the limits of the range.

### Statistical analyzes

Data were analyzed using SPSS Statistics 29 (IBM, Armonk, NY, USA). The differences in ∆Z and LD of the lesions before and after pHc were calculated by subtracting values of BL from E (∆∆Z = ∆ZE—∆ZBL). Normal distribution was assessed utilizing the Shapiro–Wilk test. Since not all groups exhibited a normal distribution, the Wilcoxon test was employed to analyze differences within individual samples. Differences between independent samples were analyzed using the Kruskal–Wallis test between groups or Mann–Whitney-U test for differences between pHc durations of groups with the same concentration. Significance level was set to *p* = 0.05. The mineral distribution within the lesions was analyzed by plotting the mean mineral content against the LD in 0.5 µm steps.

## Results

The artificial lesions at baseline (NTC_BL_) had a median (Q25/Q75) ∆Z of 7242 (6297/8405) vol% × μm and a median LD of 195 (178/221) μm. After 28 d of pHc, NTC_E_ lesions showed a significant increase in mineral density when compared to NTC_BL_ lesions (NTC_E28_: 5194 (4021/6633) vol% × μm; *p* < 0.05, Wilcoxon). However, a pHc of 56 d did not result in a significant further reduction of the integrated mineral loss compared to 28 d of pHc (NTC_E56_: 4898 (3483/6155) vol% × μm; *p* > 0.05, Mann Whitney U).

**Fig. 3:**
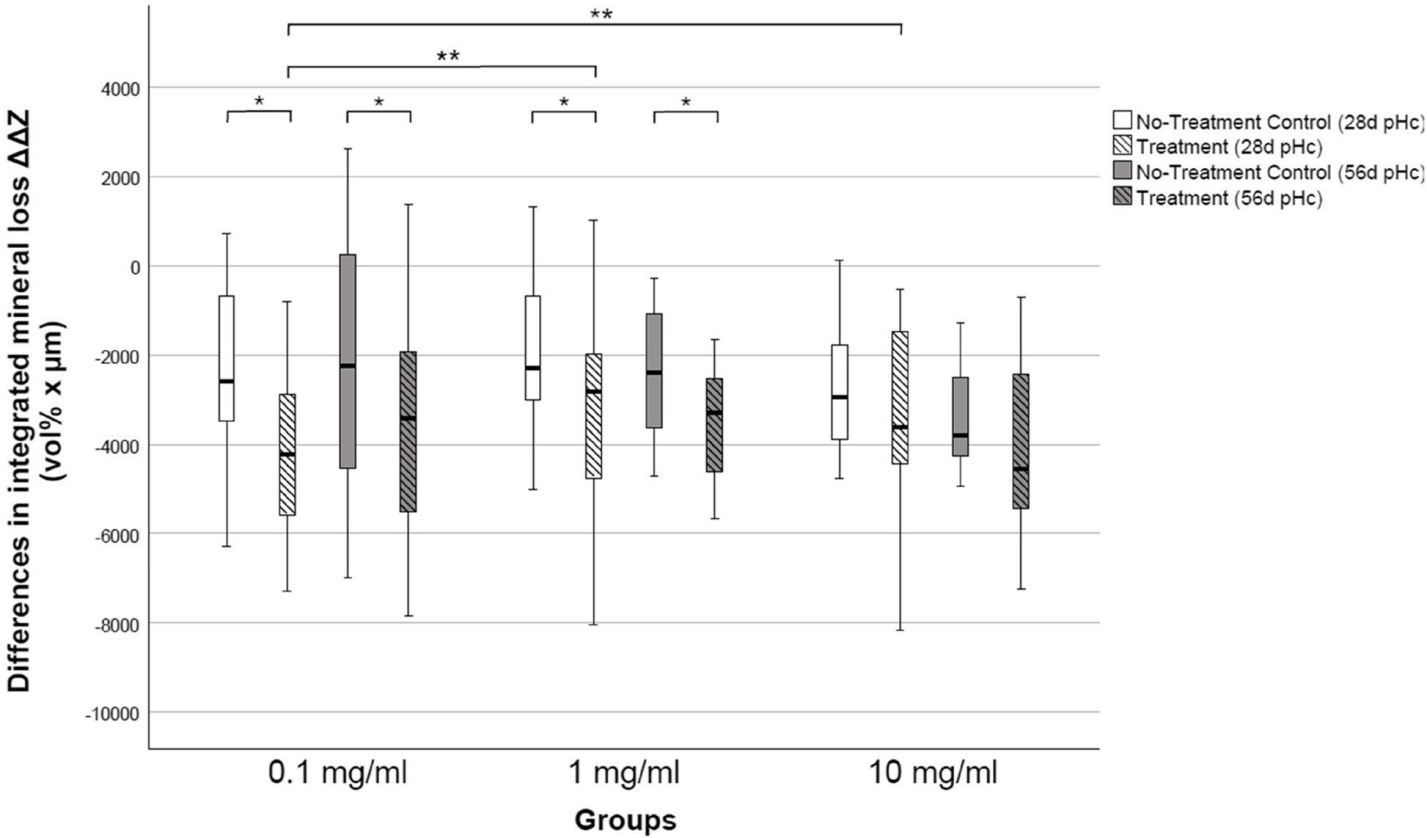
Differences in the integrated mineral loss ΔΔZ (vol% × μm) in treated (hatched) and untreated (clear) lesions after 28 d or 56 d of pHc, respectively (*n* = 17 per group). Lines: median; Boxes: 25th, 75.^th^ percentiles; whiskers: minimum–maximum. Negative values represent mineral gain. Asterisks indicate significant differences in integrated mineral loss (* *p* < 0.05, Wilcoxon; ** *p* < 0.05, Kruskal–Wallis)

Compared to NTCs, lesions treated with 0.1 and 1.0 mg/ml PAA showed a significantly higher mineral gain after 28 d of pHc (*p* < 0.05, Wilcoxon) whereas no significant differences were found for lesions treated with 10 mg/ml PAA (Table 1, Fig. 3). Furthermore, the difference in integrated mineral loss in the 0.1 mg/ml group is significantly higher compared to the difference in integrated mineral loss in the 1.0 mg/ml and 10 mg/ml group (*p* < 0.05, Kruskal–Wallis).

**Table 1:** Differences in integrated mineral loss (ΔΔZ) and lesion depth (ΔLD) in treated (T) and non-treated control (NTC) lesions after 28 d and 56 d of pHc. Values are to be reported as median and quartiles (Q25/Q75). Negative values indicate mineral gain (ΔΔZ) or lesion depth reduction (ΔLD)

PAA concentration	pHc duration	ΔΔZ (vol% × μm)		ΔLD (μm)	
		NTC	T	NTC	T
0.1 mg/ml	28d	- 2605 (- 3745/- 579)	- 4222 (- 5855/- 2781)	- 13 (- 36/19)	- 45 (- 56/- 26)
	56d	- 2252 (- 4537/254)	- 3419 (- 5525/- 1930)	- 5 (- 59/24)	- 44 (- 73/- 18)
1 mg/ml	28d	- 2294 (- 2877/- 686)	- 2826 (- 4383/- 2201)	- 4 (- 24/31)	- 33 (- 58/5)
	56d	- 2665 (- 3775/- 1371)	- 3610 (- 4616/- 2577)	- 10 (- 35/12)	- 38 (- 61/- 24)
10 mg/ml	28d	- 2952 (- 4265/- 1558)	- 3103 (- 4402/- 1051)	- 26 (- 37/0)	- 40 (- 82/- 3)
	56d	- 3806 (- 4534/- 2518)	- 4091 (- 5292/- 2207)	- 19 (- 44/28)	- 67 (- 102/- 16)

**Fig. 4:**
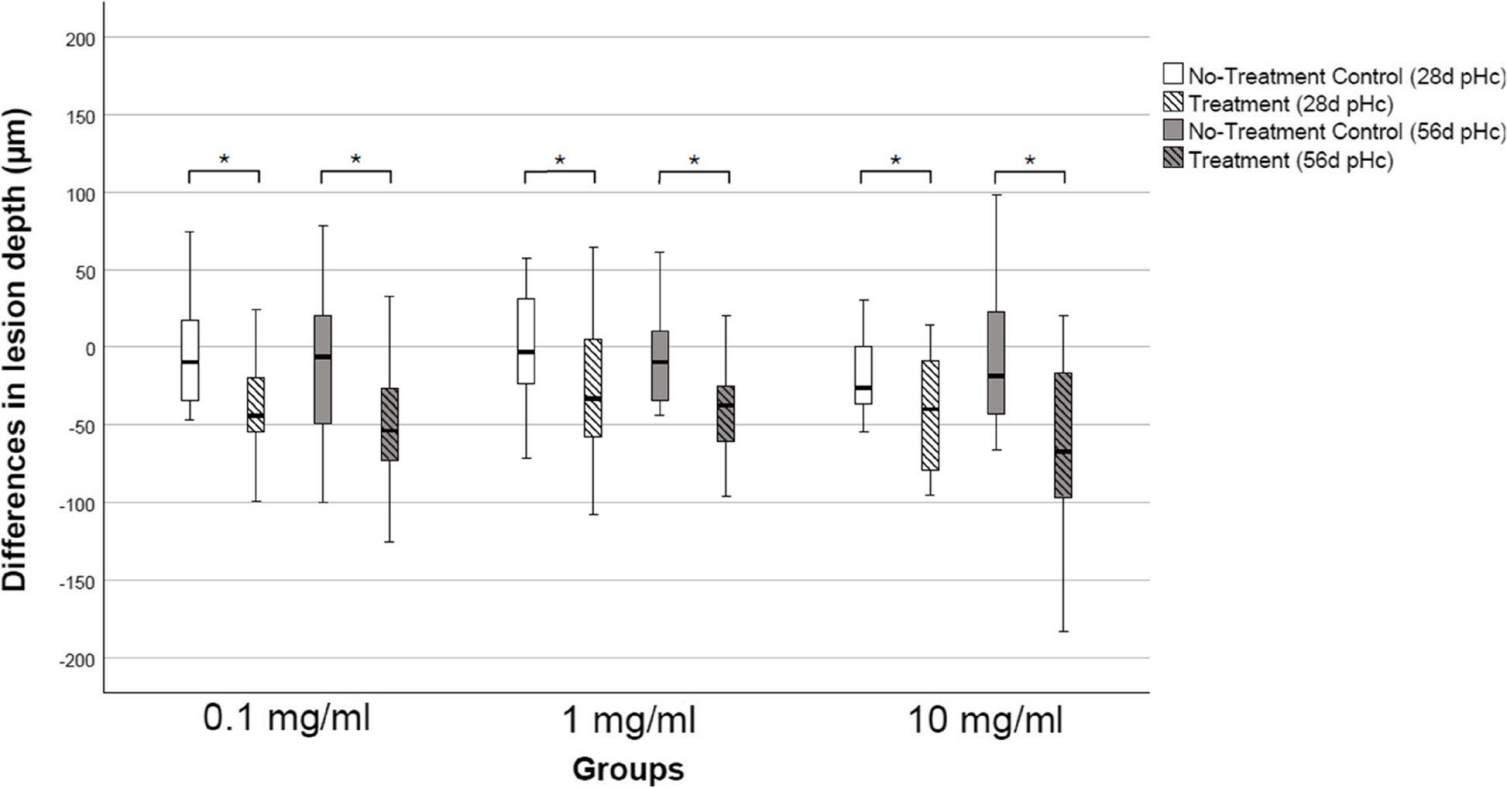
Differences in lesion depths ΔLD (μm) in treated (hatched) and untreated (clear) lesions after 28 d or 56 d of pHc respectively (*n* = 17 per group). Lines: median; Boxes: 25th, 75.^th^ percentiles; whiskers: minimum–maximum. Negative values portray the reduction in lesion depths. Asterisks indicate significant differences in lesion depths (* *p* < 0.05, Wilcoxon)

Across all groups, the extended pHc duration of 56 d did not lead to a significant further mineral gain compared to 28 d (*p* > 0.05, Mann Whitney U). Regardless the pHc time and PAA concentration, a significant decrease of LD in T lesion compared to NTC lesions was observed across all groups (*p* < 0.05, Wilcoxon) (Table 1, Fig. 4).

**Fig. 5:**
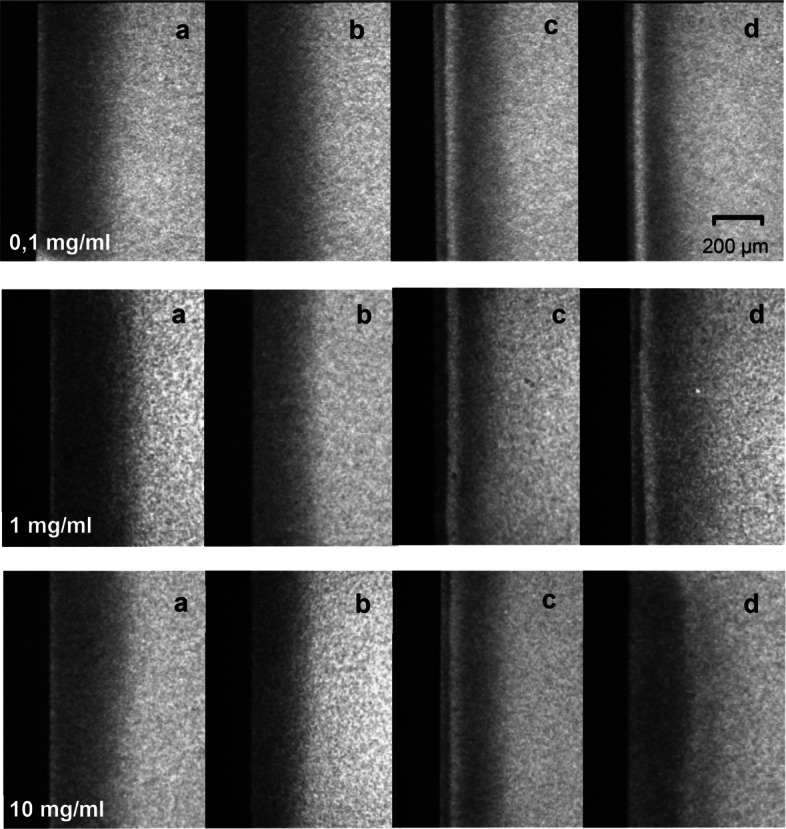
Microradiographs of corresponding lesion bodies of one specimen treated with PAA of varying concentrations. NTC_BL_ (**a**), T_BL_ (**b**), NTC_E_ (**c**) and T_E_ (**d**). In all lesions a and c, a pseudo-sound surface layer is visible, whereas in all specimens b and d, it is absent due to the etching with phosphoric acid prior to infiltration with PAA. In lesion infiltrated with PAA concentrations of 0.1 mg/ml and 1 mg/ml, a mineral band is visible within the outer half of the lesion body. In lesions treated with 10 mg/ml PAA no mineral bands were present

Microradiographs showed characteristic mineral bands in lesions after pHc. For 0.1 mg/ml PAA these bands were more pronounced compared to NTCs (Fig. 5).

**Fig. 6:**
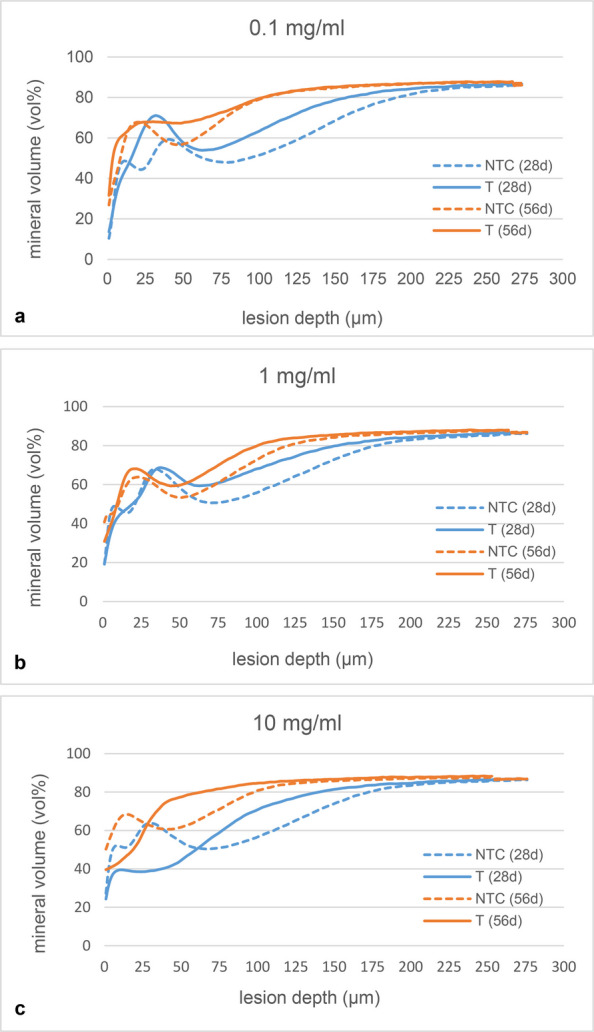
Plot of mean mineral volume throughout lesion bodies in treated (T, solid) and untreated (NTC, dashed) groups after 28 d (blue) and 56 d (orange) of pHc. In groups treated with 0.1 mg/ml (**a**) and 1 mg/ml (**b**) PAA, a mineral gain is evident throughout the lesion bodies with a peak at 25–50 µm, whereas the group treated with 10 mg/ml (**c**) exhibits a mineral loss in the outer half of the lesions and a slight mineral gain in the inner halves of the lesions

The mean mineral distribution graphs comparing treated and untreated lesions after 28 d of pHc indicate a higher mineral content throughout the lesion bodies for the treated samples with a peak between 25–50 µm for groups infiltrated with 0.1 mg/ml and 1 mg/ml PAA. In contrast, lesions treated with 10 mg/ml demonstrated a lower mineral content in the outer half of the lesions and a slightly higher mineral content in the inner part of the lesions compared to NTC (Fig. 6).

**Fig. 7:**
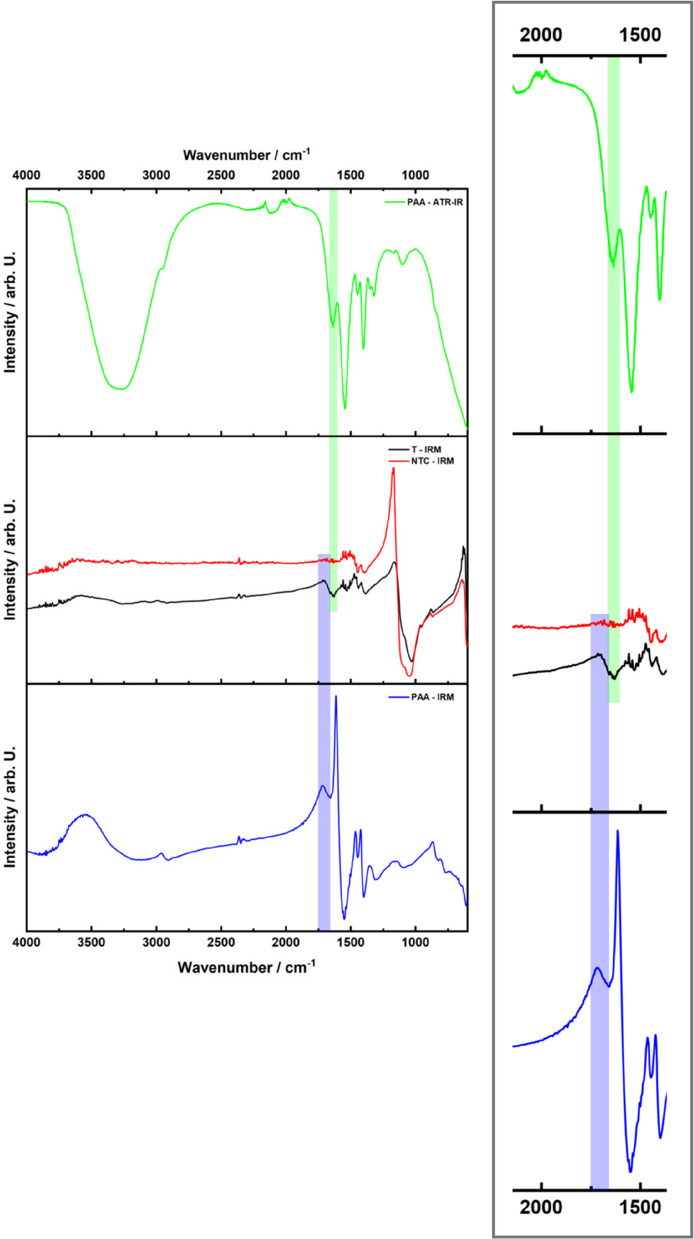
IR spectra of pure PAA (blue) as well as NTC (red) and T (black) lesions. The green spectrum shows the IR spectrum of PAA measured by an attenuated total reflection infrared (ATR-IR) spectrometer. The green and blue bar show the region of a characteristic peak of PAA measured by ATR-IR spectrometer and IRM, respectively. Only T lesions show overlapping peaks with the respective IRM and ATR-IR analysis of pure PAA in the range of 1750 cm^−1^ – 1660 cm^−1^ (R1) and 1605 cm^−1^- 1665 cm.^−1^ (R2)

**Fig. 8:**
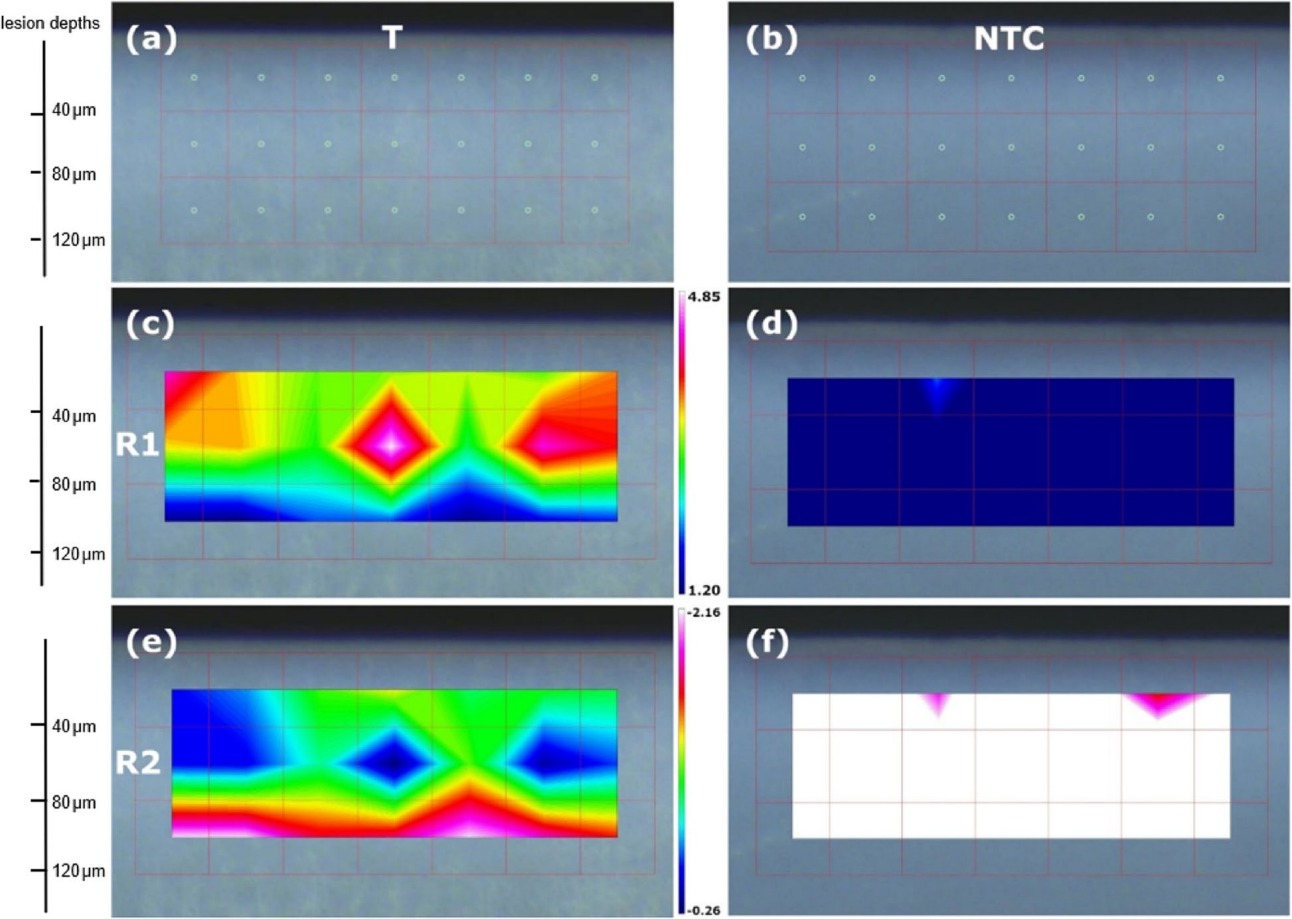
The heat maps display the integral distribution of R1 and R2 for T (**a**, **c**, **e**) and NTC (**b**, **d**, **f**) lesions in a representative sample showing the presence of PAA in T as a positive peak (reddish-pink color) for R1 and a negative peak (blueish-green color) for R2 originating as reflectance and absorbance signals from the same areas within the lesion body of the sample. Color intensity corresponds to integral magnitude. The highest visualized accumulation of PAA can be found at a lesion depth of approx. 60 μm

A comparison of the IRM spectra of the NTC and T lesions of group c revealed that only T lesions exhibited overlapping peaks with the respective IRM and ATR-IR analysis of pure PAA in the range of 1750 cm^−1^ – 1660 cm^−1^ (R1) and 1605 cm^−1^- 1665 cm^−1^ (R2) (Fig. 7). The highest signal intensity was detected at a LD of approximately 60 μm (Fig. 8).

## Discussion

The present study investigated the effects of the infiltration of artificial caries lesions with PAA solutions in different concentrations in a pHc model. We found significant differences in the integrated mineral loss between different PAA concentrations. However, the prolongation of the subsequent pHc did not result in a further increase in mineral gain in either the control or the treated lesions. Therefore, our null hypothesis can be partially rejected. Following prior findings demonstrating the remineralizing effects of PAA on early carious lesions [19], we additionally aimed to verify the infiltration of PAA into the lesion bodies. Using IRM, we could confirm the presence of PAA in the lesion bodies exclusively in infiltrated lesions, proving successful loading with PAA.

Various remineralization agents have been investigated for their potential to biomimetically restore enamel tissue in early caries lesions [13, 34–37]. The primary objective remains to promote the formation of apatite within the lesion body. However, this process presents significant chemophysical challenges. For example, early caries lesions present natural barriers, such as a highly mineralized surface layer, which limits remineralization of subsurface lesion areas. To tackle this limitation, this study employed the concept of infiltration to initiate biomimetic remineralization through polymer-induced liquid precursors in the lesion bodies. This pathway of non-classical crystallization has been shown to promote mineralization in collagen-containing hard tissues [23–25]. Previous studies indicate that the concentration to plays a key role in PAA-driven crystallization dynamics [26, 29–31, 38].

In our experimental setup, lesions treated with 0.1 mg/ml PAA showed significantly greater mineral gain than specimens treated with 1 mg/ml and 10 mg/ml PAA. With varying concentrations, the ratio of carboxylate groups to Ca^2+^ changes. This ratio has been shown to impact the crystallization by influencing the stability of calcium carbonate complexes, which has been identified as a pivotal factor in the regulation of crystal formation and aggregation [27, 28]. Previous studies that investigated the impact of polymer concentration in collagenous tissue demonstrated that the interplay between inhibiting and promoting crystallization processes with different concentrations can be leveraged to facilitate remineralization in bone and dentine [29–31]. The addition of PAA in higher concentrations, and thus with a higher carboxylate groups to Ca^2+^ ions ratio, results in the formation of more stable amorphous calcium phosphate complexes that are less likely to undergo conversion to HAP due to kinetic energy factors [29, 31]. This aligns with our findings, which indicate that lower concentrations of PAA tend to promote greater mineral gain. The ratio of available reactive groups relative to Ca^2+^ ions further appears to affect the morphology of the liquid precursors. It has been demonstrated that PAA at lower concentrations leads to a sharp initial rise in pH through the rapid release of OH- as a byproduct, thereby causing the polymer chains to stretch and form elliptical calcites. Conversely, high PAA concentrations have been shown to induce a gradual pH increase, leading to spherical precursors [28, 39]. Wang et. al reported that PAA at a concentration of 0.5 mg/ml produced loosely organized clusters resembling PILPs that are capable of penetrating deeper areas of collagen matrix and induce crystallization processes through aggregation—one proposed pathway of NCC. At higher concentrations of 1.0 mg/ml, however, PAA formed isolated prenucleation clusters which have shown to retard phase transformation and not aggregating to crystals [31]. These physical properties may also influence the infiltration process in the present study, as smaller, more liquid structuring units are likely more effective at penetrating demineralized enamel and reaching deeper lesion areas to initiate remineralization. Consequently, further experiments may be conducted to test even lower concentrations of PAA (e.g., < 0.1 mg/ml) for enamel lesions to identify the optimal dosage range, wherein the total number of carboxylic groups is sufficient to form PAA-Ca^2+^ complexes, yet not so high as to excessively stabilize these complexes and thereby impede HAP-mineralization processes. The application time of 10 min has been chosen as it showed a visible infiltration (the drops disappearing) and corresponds to the rather explorative character of this study. Further studies could explore if shorter exposure periods deliver similar effects and therefore providing a better clinical feasibility.

The IRM analyses were conducted with the highest concentration of PAA employed in this experimental setup (10 mg/ml), as it yielded the highest measurable signal and thus served as a proof of principle. Due to the nature of IRM, absorbance and transmission signals may overlap with reflection signals. Absorbance and transmission signals are exclusively positive or negative, respectively, whereas reflection signals can exhibit both positive and negative maxima relative to the baseline [40, 41]. The double peak (R1, R2) observed in the T lesion spectrum could therefore be attributed to a reflection signal. The cluster-like distribution may be interpreted as the formation of PAA/Ca^2+^ complexes acting as liquid precursors, serving as structuring units for crystal growth – an effect consistent with previously reported mechanisms of NCC [20, 21, 42, 43].

Extending the pHc period to 56 days did not result in significantly higher remineralization compared to a 28-day cycle in both treated and untreated groups. In this experimental setup, PAA was applied only once prior to the pHc, providing only a limited supply of reactive carboxylate groups capable of binding Ca^2^⁺ ions, which may have led to an early saturation of liquid precursors required for ongoing mineralization. The remineralization phase of the pHc was carried out with the incorporation of fluoride to enhance its transferability in clinical settings, as fluoride is recognized as the most prevalent remineralization agent. It can be assumed that prolonged pHc periods combined with a constant supply of fluoride ions promote the formation of a more highly mineralized surface layer as well as previously described subsurface mineral bands [44, 45]. In all lesions that underwent pHc a characteristic mineral band underneath the surface layer was observed, most likely resulting from the presence of fluoride ions in the pHc as previously reported [44–46]. This formation is believed to be associated with altered diffusion and buffering properties due to the formation of large fluorapatite and fluorhydroxyapatite crystallites [47]. Notably, these mineralized bands were more pronounced in lesions treated with lower PAA concentrations, clearly diminishing with increasing concentrations and even completely vanishing in lesions infiltrated with 10 m/ml PAA (Fig. 5). This observation supports our previously stated hypothesis that PAA-driven crystallization promotes mineral retention in deeper lesion areas, with the present study highlighting the concentration-dependent nature of this effect. Furthermore, these mineral bands were located approximately 50 µm below the lesion surfaces, corresponding to the region where IRM analysis showed the highest accumulation of PAA (Fig. 8). This, in turn, may restrict the diffusion of Ca^2^⁺ ions into subsurface areas, thereby limiting the availability of amorphous calcium phosphates for HAP formation. The absence of mineral bands in lesions infiltrated with a high concentration of 10 mg/ml PAA suggests inhibiting interactions between PAA and fluoride that hinder both the development of mineral bands as well as mineralization within deeper lesion areas. It might be speculated if repeated PAA applications in order to facilitate deeper infiltration, reintroduce reactive carboxylate groups and potentially enhance remineralization within the lesion bodies over extended pHc periods.

This study has several limitations. The specimens were prepared from bovine incisors due to their convenient acquisition in large quantities, significantly larger surfaces and more uniform composition than human teeth, proven to be a suitable surrogate for mineralization experiments [48, 49]. As in previous studies, a (resin) infiltration protocol was implemented in the present study [19, 50–52]. This protocol had been developed to remove the pseudo-intact surface layer and desiccate the porous lesion body in order to establish the necessary conditions for an effective infiltration of the PAA into the lesion bodies of the artificial caries lesions using capillary forces [53, 54]. An application of PAA without the removal of the pseudo-intact surface layer may have resulted in mineral gain confined to the surface layer, due to early aggregation of calcium and phosphate. Natural caries lesions, however, typically exhibit a broader spectrum of characteristics, including variations in the (usually considerably more pronounced) surface layer thickness and significantly greater lesion depths. Consequently, clinical treatment protocols generally involve the erosion of lesion surfaces with more potent acids such as hydrochloric acid over extended periods [55]. As a result, our in vitro setting inherently lacks direct applicability to clinical conditions. Another limitation pertains to the disparate lesion management of NTC and T lesions, with treated lesions being etched prior to pH-cycling and non-treated controls not undergoing etching. This discrepancy may hinder the differentiation between the remineralizing effects of PAA and mineralization induced by the pHc. Nonetheless, a decision was made to compare the infiltration treatment to true non-treatment controls, aiming to enhance potential applicability to clinical practice.

In order to replicate the natural process of pH changes in the oral cavity and to provide a sufficient and constant source of mineral ions, a pHc model with rather remineralizing conditions was employed. While pHc models are a valuable tool to simulate oral conditions of fluctuating hyper- and hypo-saturated conditions, they remain a simplification of intraoral processes, including the presence of bacterial biofilms or natural saliva [56–58]. However, pHc models still enable high levels of experimental control and reproducibility for single variable studies.

Finally, the present study does not provide information on the long-term stability of the mineralization products within caries lesions, particularly under extended time frames or renewed demineralizing conditions. Future investigations should not only address these aspects, but also explore the effects of varying PAA chain lengths, as these alter the ratio of carboxylate groups to Ca^2^⁺ ions and may thereby influence the dynamics of mineralization [28, 29]. This experimental setup served as a proof of concept to verify the remineralization potential of PAA. Follow-up studies could also include comparisons with commonly used treatment approaches of white lesions such as fluoride varnishes or CPP-ACP.

## Conclusion

Within the limitations of this in vitro study it can be concluded that artificial caries lesions can be effectively infiltrated with PAA solutions. Under remineralizing pH-cycling conditions, lower concentrations of PAA seem to promote mineral gain within the lesion body. A prolonging pH-cycling time does not seem to further enhance this effect.

## Data Availability

The data to support the findings of this study are available from the corresponding author upon reasonable request.

## Abbreviations

PAA: Polyacrylic Acid
pHc: PH-cycling
NTC: Non-treatment control
BL: Baseline
E: Effect
T: Treated
ML: Mineral loss
LD: Lesion depth
IRM: Infrared microscopy
